# The evolutionary trade‐off between stem cell niche size, aging, and tumorigenesis

**DOI:** 10.1111/eva.12476

**Published:** 2017-04-14

**Authors:** Vincent L. Cannataro, Scott A. McKinley, Colette M. St. Mary

**Affiliations:** ^1^ Department of BiostatisticsYale School of Public HealthYale UniversityNew HavenCTUSA; ^2^ Department of BiologyUniversity of FloridaGainesvilleFLUSA; ^3^ Department of MathematicsTulane UniversityNew OrleansLAUSA

**Keywords:** aging, biomedicine, evolutionary theory, population dynamics, population genetics—theoretical, stem cells, tumorigenesis

## Abstract

Many epithelial tissues within multicellular organisms are continually replenished by small independent populations of stem cells largely responsible for maintaining tissue homeostasis. These continually dividing populations are subject to mutations that can lead to tumorigenesis but also contribute to aging. Mutations accumulate in stem cell niches and change the rate of cell division and differentiation; the pace of this process and the fate of specific mutations depend strongly on niche population size. Here, we create a mathematical model of the intestinal stem cell niche, crypt system, and epithelium. We calculate the expected effect of fixed mutations in stem cell niches and their effect on tissue homeostasis throughout the intestinal epithelium over organismal lifetime. We find that, due to the small population size of stem cell niches, mutations predominantly fix via genetic drift and decrease stem cell fitness, leading to niche and tissue attrition, and contributing to organismal aging. We also explore mutation accumulation at various stem cell niche sizes and demonstrate that an evolutionary trade‐off exists between niche size, tissue aging, and the risk of tumorigenesis. Further, mouse and human niches exist at a size that minimizes the probability of tumorigenesis, at the expense of accumulating deleterious mutations due to genetic drift. Finally, we show that the trade‐off between the probability of tumorigenesis and the extent of aging depends on whether or not mutational effects confer a selective advantage in the stem cell niche.

## 1 Introduction

Multicellular organisms continually accumulate mutations within their somatic tissues, constituting a significant, but poorly quantified, burden on tissue maintenance. To investigate this burden in a specific, well‐parameterized context, we model the mammalian intestine and quantify the expected impact of mutation accumulation in stem cell populations. For this analysis, we build on our existing model of mutational dynamics in intestinal crypts (Cannataro, McKinley, & St. Mary, 2016) and recently elucidated population dynamics of intestinal stem cells and stem cell progeny (Baker et al., 2014; Kozar et al., 2013; Ritsma et al., 2014). Furthermore, we explore how the population size of the stem cell niche influences mutation accumulation and demonstrate the expected trade‐off between the risk of accumulating deleterious mutations, population size, and the risk of tumorigenesis (Michor, Iwasa, Komarova, & Nowak, 2003). However, we further characterize how this trade‐off can be expected to manifest over the lifetime of two well‐studied mammalian systems, mice and humans, by estimating the expected effect of mutation accumulation on cellular homeostasis.

The paradigm of stem cell turnover within the intestine is not unique to that tissue (Klein & Simons, 2011), and thus, the work presented here can provide insights in other systems undergoing continual self‐renewal through the division of stem cells.

### 1.1 Deleterious mutations accumulate throughout the intestinal epithelium

The intestinal epithelium is in constant flux, with populations of stem cells distributed throughout the intestine differentiating into other, transient, cell populations (Morrison & Spradling, 2008). These stem cells exist within small discrete populations, a compartmentalization thought to have evolved as a mechanism to deter tumorigenesis, as cells accumulating mutations that are beneficial to cellular fitness have a physical hindrance to spreading throughout the tissue (Cairns, 1975). However, small populations are subject to significant genetic drift, that is, random changes in allele frequency that eventually lead to fixation or loss, and less effective selection. Furthermore, due to the asexual nature of mitotic division and stem cell maintenance, stem cell populations are a prime example of Muller's ratchet, or, the irreversible accumulation of mutations and resultant decrease in a population's mean fitness in the absence of recombination (Lynch, Burger, Butcher, & Gabriel, 1993; Muller, 1964). Under normal circumstances, mutations are predicted to occur throughout the intestines (Lynch, 2010), and the majority of mutations that affect fitness will decrease fitness (Eyre‐Walker & Keightley, 2007). In populations of whole organisms, especially those in which fitness effects are studied in the laboratory, these mutations rarely become fixed due to large population sizes and purifying selection (see Levy et al. (2015) for a recent example). We have previously estimated that crypts are predominantly accumulating deleterious mutations over the lifetime of the host organism (Cannataro et al., 2016). Here, we quantify the expected change in whole‐tissue equilibrium population size as mutations accumulate in the stem cell populations at the base of intestinal crypts over an organism's lifetime.

### 1.2 Fixed deleterious mutations within somatic tissue contribute to organismal aging

The accumulation of damage causing the loss of cellular fitness is a hallmark of aging and is especially relevant when DNA damage occurs in stem cells, compromising their role in tissue renewal (López‐Otín, Blasco, Partridge, Serrano, & Kroemer, 2013). Indeed, several mouse models with the diminished ability to maintain cellular genome integrity succumb to accelerated age‐related phenotypes through the loss of tissue homeostasis caused by stem and progenitor cell attrition (Ruzankina, Asare, & Brown, 2008). Just as stem cell mutations conferring a beneficial fitness effect will increase cell production, mutations conferring a deleterious fitness effect will lead to decreased cell production and the diminished maintenance of healthy tissue. Stem cells at the base of the intestinal crypt differentiate into all other intestinal cell populations (Barker, 2014). Hence, mutations affecting the rates of stem cell dynamics will propagate through other populations, affecting their steady‐state equilibrium population sizes. We model the various cell populations of the intestinal crypt and epithelium to calculate how mutations occurring in stem cell lineages govern population dynamics throughout the tissue.

### 1.3 Stem cell niche population size influences the rate of aging and tumorigenesis

Unique and previously unexplored evolutionary pressures exist within somatic tissue. It has been shown that small stem cell populations may promote genetic instability by allowing mutator mutations to fix via drift, a process that would increase the rate of tumorigenesis within a crypt as niche population size decreased (Michor et al., 2003). This, coupled with the fact that the strength of selection increases with increasing population size, suggests that there exists an intermediate stem cell niche size that minimizes the probability of fixing mutations of large beneficial effect that lead to tumorigenesis. Additionally, the large intrinsic rate of deleterious mutations, coupled with the weak selective pressure at small population sizes that allows deleterious mutations to drift to fixation, suggests that small population sizes will result in the extensive accumulation of deleterious mutations and tissue attrition. Thus, population sizes that minimize the chance of tumor formation throughout a tissue may do so at the expense of accumulating deleterious mutations, a process that contributes to aging, resulting in an evolutionary trade‐off between aging and tumorigenesis. We estimate the probability of tumorigenesis within a single crypt as a function of stem cell niche size, under normal mutation accumulation expectations and two different selective regimes, and compare the results to our estimates of whole‐tissue equilibrium size change to explore the evolutionary trade‐off between stem cell niche size, organismal aging, and tumorigenesis.

## 2 Methods/Modeling

We are interested in how the rates of stem cell division and differentiation scale up to whole‐tissue dynamics. Within this section, we first describe the general architecture of crypt systems. Then, we detail our parameterization of the models in light of recent experiments in mice. Finally, we describe how we model the evolutionary processes occurring within crypts and throughout the tissue.

### 2.1 Modeling the crypt system

#### 2.1.1 Intestinal crypts are composed of the various types of cell populations derived from stem cells

**Figure 1:**
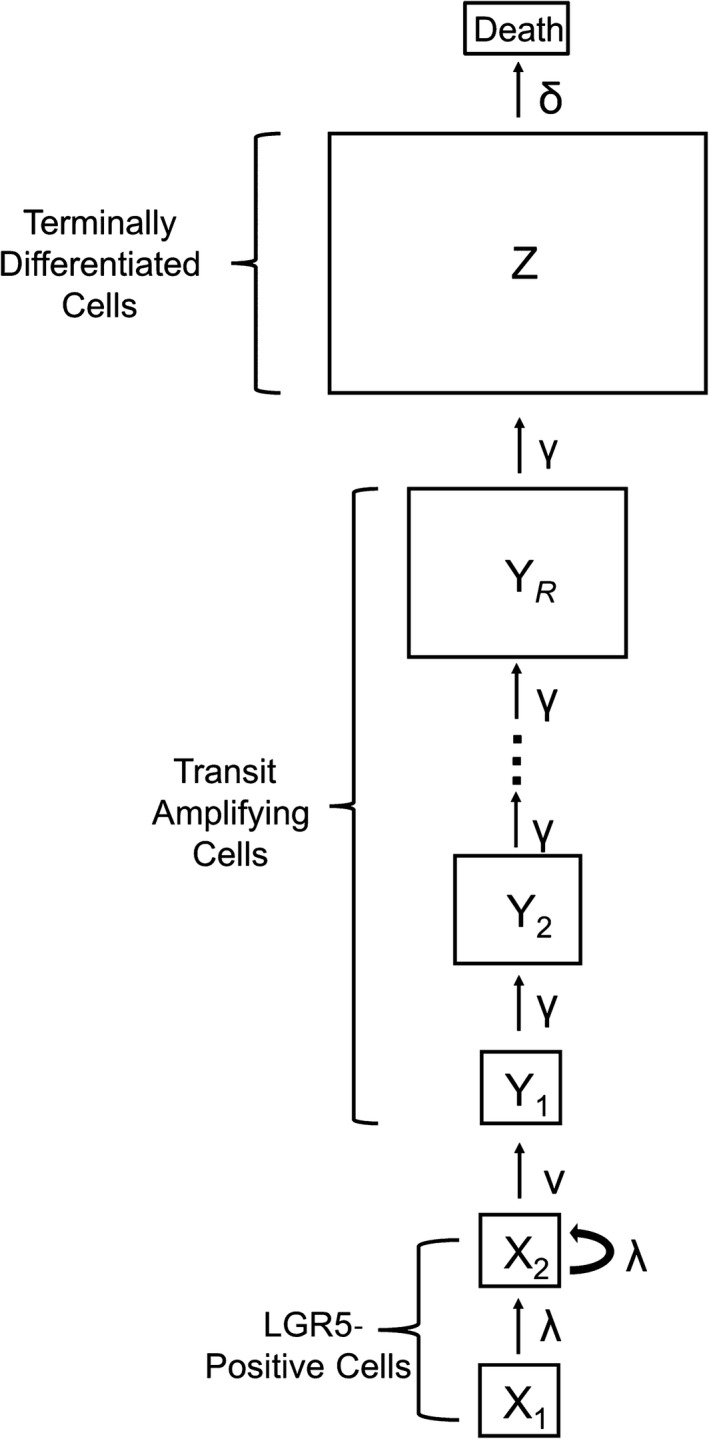
The general architecture of a crypt system. Population names are within the boxes and the rates at which cells accumulate within or are transferred between populations are next to the arrow portraying their transition

Mouse crypts contain a population of self‐renewing LGR5^+^ cells at their base. LGR5^+^ cells continuously divide and differentiate into all epithelial cells within the intestinal epithelium (Barker et al., 2007), and a single LGR5^+^ cell can form crypt–villus systems in vitro (Sato et al., 2009). Thus, by demonstrating both self‐renewal capabilities and multipotency, the LGR5^+^ populations are considered the putative stem cell populations of the intestinal epithelium. Within this population, there exists a functional subpopulation responsible for maintaining homeostasis within the crypt (Baker et al., 2014; Kozar et al., 2013). We call this subpopulation responsible for crypt homeostasis *X*_1_, and these cells inhabit the stem cell niche. The cells in the stem cell niche divide symmetrically and undergo neutral drift, where any lineage with the same division rate as the other lineages in the crypt has equal probability to reach monoclonality by displacing all other lineages through division into a stem cell population bordering the transit‐amplifying population we call *X*_2_. These displaced cells may also divide symmetrically or commit to differentiation (Ritsma et al., 2014). Cells with greater division rate have an increased probability of displacing their neighbors; those with lower division rate are more likely to be displaced. In other words, niche cells that divide more rapidly have a selective advantage. In our model, both stem cells within the niche and displaced stem cells divide symmetrically at rate λ, and displaced stem cells commit to differentiation at rate ν and join the transit‐amplifying (TA) compartment, *Y*_1_, located above the stem cell compartment. A TA cell rapidly divides at rate γ a number of rounds, *R*, joining subsequent TA pools (*Y*_1_, *Y*_2_, …, *Y*_*R*_). After the last round, the cell divides a final time and joins the terminally differentiated postmitotic cell pool *Z* (Potten, 1998). Cells within the postmitotic cell pool exist until they undergo apoptosis at rate δ either at the villus tip or lumenal surface in the small intestine and large intestine, respectively (Grossmann et al., 2002). The terminally differentiated cells maintain the functionality of the intestinal tissue, with many existing at the top of the crypt, on the epithelial surface lining the lumen, and, in the case of the small intestine, along the villi. The dynamics described above are depicted in Figure 1.

These dynamics are represented by the transition rates

$$\begin{array}{cc} & (Z)\to (Z-1)\;\text{at}\text{rate}\;\delta Z,\\ & \left(Y_{R},\;Z\right)\to \left(Y_{R}-1,\;Z+2\right)\;\text{at}\text{rate}\;\gamma Y_{R},\\ & \vdots \\ & \left(Y_{2},Y_{3}\right)\to \left(Y_{2}-1,Y_{3}+2\right)\;\text{at}\text{rate}\;\gamma Y_{2},\\ & \left(Y_{1},Y_{2}\right)\to \left(Y_{1}-1,Y_{2}+2\right)\;\text{at}\text{rate}\;\gamma Y_{1},\\ & \left(X_{2},Y_{1}\right)\to \left(X_{2}-1,Y_{1}+1\right)\;\text{at}\text{rate}\;\nu X_{2},\\ & \left(X_{2}\right)\to \left(X_{2}+1\right)\;\text{at}\text{rate}\;\lambda X_{2},\\ & \left(X_{1},X_{2}\right)\to \left(X_{1},\;X_{2}+1\right)\;\text{at}\text{rate}\;\lambda X_{1}. \end{array}$$

The time‐dependent means of this system satisfy the ordinary differential equations:

(1) $$\begin{array}{cc} & \frac{d}{dt}\overset{¯}{z}\left(t\right)=2\gamma {\overset{¯}{y}}_{R}\left(t\right)-\delta \overset{¯}{z}\left(t\right),\\ & \frac{d}{dt}{\overset{¯}{y}}_{R}\left(t\right)=2\gamma {\overset{¯}{y}}_{R-1}\left(t\right)-\gamma {\overset{¯}{y}}_{R}\left(t\right),\\ & \vdots \\ & \frac{d}{dt}{\overset{¯}{y}}_{2}\left(t\right)=2\gamma {\overset{¯}{y}}_{1}\left(t\right)-\gamma {\overset{¯}{y}}_{2}\left(t\right),\\ & \frac{d}{dt}{\overset{¯}{y}}_{1}\left(t\right)=\nu {\overset{¯}{x}}_{2}\left(t\right)-\gamma {\overset{¯}{y}}_{1}\left(t\right),\\ & \frac{d}{dt}{\overset{¯}{x}}_{2}\left(t\right)=\lambda {\overset{¯}{x}}_{1}\left(t\right)+\lambda {\overset{¯}{x}}_{2}\left(t\right)-\nu {\overset{¯}{x}}_{2}\left(t\right),\\ & \frac{d}{dt}{\overset{¯}{x}}_{1}\left(t\right)=0,\;\mathrm{we}\mathrm{model}\;X_{1}\;\mathrm{as}a\mathrm{fi}\;\mathrm{xed}\mathrm{population}\mathrm{size}. \end{array}$$

Setting the left‐hand sides for each equation in the system (1) to zero, we can solve for the steady‐state mean of the terminally differentiated population, Z*. We find that Z* can be expressed in terms of the system's rate parameters and the stem cell niche population size *X*_1_. The steady‐state Z* (Equation 2) is a function of all of the rate parameters in the system except the TA cell division rate:

(2) $$Z^{*}=\frac{2^{R}\nu \lambda X_{1}^{*}}{\delta (\nu -\lambda )}.$$

This function is used in subsequent analyses to calculate how mutations to the various rate parameters change the tissue population size. Of note, a prediction of this model is that a mutation to either division rate or differentiation rate will result in an amplified effect on the proportion of change in steady‐state postmitotic cells. That is, if λ_0_ is mutated to λ_1_, the proportional difference in the postmitotic cell population is $\frac{Z_{1}^{*}}{Z_{0}^{*}}=\frac{\lambda_{1}}{\lambda_{0}}\times \frac{\left(\nu -\lambda_{0}\right)}{\left(\nu -\lambda_{1}\right)}$. The population maintains a steady‐state population size when the differentiation rate is greater than the division rate.

### 2.2 Model parameterization

#### 2.2.1 Parameterizing the crypt model using mouse small intestine data

The most studied crypt system is in the mouse small intestine. There are 14–16 LGR5^+^ putative stem cells in the base of the mouse small intestinal crypts, and it is believed that they divide symmetrically under neutral competition (Lopez‐Garcia, Klein, Simons, & Winton, 2010; Snippert et al., 2010). Kozar et al. (2013) found that there exists a subset of this population that maintains crypt homeostasis and that this subpopulation consists of approximately five, six, and seven cells dividing approximately 0.1, 0.2, and 0.3 times per day along the proximal small intestine, distal small intestine, and colon, respectively. When estimating the expected effects of mutation accumulation along the entire intestine in the mouse, we take the median value of cell number and division rate found by Kozar et al. (2013) to be the number of cells in the niche, *X*_1_, and the stem cell symmetric division rate, λ, with the remainder of the stem cell population constituting the *X*_2_ population. That is, the “working” stem cells that are responsible for homeostasis (Kozar et al., 2013) and displace each other through symmetric division (Ritsma et al., 2014) exist in *X*_1_, while cells displaced into *X*_2_ both divide symmetrically and commit to differentiation. To maintain the number of LGR5^+^ stem cells at a steady state of 15, we calculate a rate of committing to differentiation ν of 0.333 per day. Transit‐amplifying cells divide twice a day (Potten, 1998; Potten & Loeffler, 1987) and undergo approximately six generations of amplification, with each stem cell eventually contributing about 64 cells to the crypt system (Marshman, Booth, & Potten, 2002). These lineages end at their terminally differentiated cell stage and live for 2–3 days before dying and leaving the intestine (Snippert et al., 2010). The parameters described above are provided in Table 1.

**Table 1:** Approximate parameter values for the mouse intestine

Parameter	Value	Note	Citation
*X*_1_	6 Cells	Expected number of stem cells in the small intestine stem cell niche	Kozar et al. (2013)
$X_{1}+X_{2}^{*}$	15 Cells	The total number of LGR5^+^ in the base of the crypt	Snippert et al. (2010) and Clevers (2013)
λ	0.2 per day	The division rate of the subpopulation of the stem cells constituting the niche	Kozar et al. (2013)
ν	0.333 per day	The rate at which cells passively adopt a TA fate	This study
γ	2 per day	The division rate of TA cells	Potten and Loeffler (1987)
δ	0.333 per day	The death rate of terminally differentiated cells	Snippert et al. (2010)
*R*	6	Rounds of division of rapidly dividing TA cells	Marshman et al. (2002)

Parameterizing our structured model with the values from Table 1, the model generates observed cell pool population sizes. Using the model, we calculate the steady‐state mean population sizes of the various TA populations to be $\left(Y_{1}^{*},Y_{2}^{*},Y_{3}^{*},Y_{4}^{*},Y_{5}^{*},Y_{6}^{*}\right)=\left(1.5,3,6,12,24,48\right)$. This results in the total rapidly dividing TA population being $Y_{\mathrm{total}}^{*}\approx 95$ cells, slightly underestimating estimates from the literature of the number of cells within this compartment which are around 120 (Marshman et al., 2002). These dynamics result in a steady‐state mean of the terminally differentiated cell population size in our model, Z*, to equal 576 cells. The crypts consist of approximately 250 cells (Potten & Loeffler, 1990), meaning that (accounting for the 95 TA cells, the 15 stem cells, and the 10 Paneth cells not modeled here; Clevers, 2013) the terminally differentiated cell population in our model exists as 130 cells within the crypt and 466 cells outside of the crypt. The villi in the small intestine are supported by six to 10 crypts and consist of approximately 3,500 cells (Potten & Loeffler, 1990). The total steady‐state mean population in our model contributed by eight crypts to a villus using the parameters in Table 1 is 8 × 446 = 3,568 cells.

We are interested in estimating the effects of mutation accumulation throughout the whole intestinal tract in the mouse; hence, we take the median value of certain parameters that vary from proximal small intestine to colon (as described above). We note that if one was interested in the effects of steady‐state terminally differentiated population size in just the proximal small intestine or colon, this analysis may overestimate or underestimate the effects, respectively, as there are more stem cells dividing faster in the colon than the proximal small intestine. Additionally, the large intestine TA cells may undergo more rounds of division than the small intestine TA cells (Potten, 1998).

#### 2.2.2 Intrinsic mutational effects

The entire distribution of fitness effects of mutations is unknown for somatic tissues. Additionally, although the mutation rate has been estimated on a per‐nucleotide basis (Jones et al., 2008), the mutational target size of all mutations that affects crypt cell fitness is unknown. Thus, we explore the implications of a wide range of intrinsic mutational parameters within somatic tissue. However, despite the differences between the genomes of species, mutation accumulation experiments and analysis of DNA sequence data have revealed general principles regarding distributions of intrinsic mutational effects, namely mutations are much more likely to confer a deleterious fitness effect than a beneficial effect, and that the deleterious effect will have a larger expected value (Bank, Hietpas, Wong, Bolon, & Jensen, 2014; Eyre‐Walker & Keightley, 2007). Deleterious fitness effects are extremely difficult to characterize in typical laboratory experiments because selection against deleterious mutations is effective in even moderately sized populations (Estes, Phillips, Denver, Thomas, & Lynch, 2004; Keightley & Halligan, 2009). Directed mutagenesis experiments are one way to classify the distribution and average effect of mutations deleterious to fitness. For instance, Sanjuán, Moya, and Elena (2004) performed site‐specific single‐nucleotide substitutions in an RNA virus and found that the average deleterious nonlethal fitness effect decreased growth rate by approximately 20%. Similarly, when grown in permissive conditions that allow spontaneous deleterious mutations to accumulate through genetic drift, Keightley and Caballero (1997) found a 21% average deleterious fitness effect per mutation in *Caenorhabditis elegans* and Zeyl and DeVisser (2001) found a 21.7% average fitness decline per fixed mutation in diploid strains of the single‐celled eukaryote *Saccharomyces cerevisiae*. Other mutation accumulation studies have found more modest deleterious effects, such as the average fitness decline in haploid *S. cerevisiae* per mutation of 8.6% found by Wloch, Szafraniec, Borts, and Korona (2001). Another mutation accumulation experiment in *S. cerevisiae* found the expected beneficial increase in fitness per mutation to be 6.1%, the rate of mutation that affects fitness per mutation to be 1.26 × 10^−4^, and the percent of fitness effects that are beneficial to be 5.75% (Joseph & Hall, 2004). When our analysis requires specific parameter choices, as in Section 3.3 when we juxtapose the dynamics of mutations that fix neutrally with those under selection, we utilize the *S. cerevisiae* parameters described here, but note that we are interested in characterizing the dynamics of tumorigenesis and aging, and we are not making conclusions about the absolute magnitude of either given the limited knowledge of mutational effects in somatic tissue.

### 2.3 Modeling evolution within somatic tissue

#### 2.3.1 Modeling the expected mutational effect of a single mutation within a crypt

To quantify the expected effect on tissue homeostasis of mutations in epithelial tissue, it is necessary to understand the processes of mutation accumulation and fixation within the stem cell niche populations at the base of the intestinal crypts. Mutations in the niche can be placed into two different categories: mutations that directly affect the stem cell phenotype associated with cellular fitness, that is, division rate, within the stem cell niche, and mutations that do not affect the fitness of stem cells within the niche. Mutations that affect the division rate of stem cells will confer a fitness advantage or disadvantage because it is the symmetric division of stem cells into more stem cells that determines the rate a lineage replaces its neighbors and fixes in the population. For instance, certain mutations to KRAS increase stem cell division rate and the probability this mutant lineage reaches fixation (Snippert, Schepers, van Es, Simons, & Clevers, 2014; Vermeulen et al., 2013). Mutations that do not directly affect stem cell division rate will not alter stem cell fitness, because they do not affect the cell phenotype while it is within the niche and will fix neutrally.

We model the distribution of mutational effects and mutation accumulation similarly as in Cannataro et al. (2016), where we provide a detailed mathematical methodology. Briefly, mutational effects are distributed exponentially, with expected deleterious effect *s*_−_, expected beneficial effect *s*_+_, and *P*_B_ percent of mutations conferring a beneficial effect. Thus, mutational effects on the initial stem cell division rate, λ_0_, are distributed according to Equation (3), where

(3) $$m{\left(\lambda ;\lambda_{0}\right)}_{\exp}=\left\{\begin{array}{cc} \left(1-P_{B}\right)\frac{\beta }{\lambda_{0}}e^{-\beta \left(1-\frac{\lambda }{\lambda_{0}}\right)} & \lambda <\lambda_{0}\\ P_{B}\frac{\alpha }{\lambda_{0}}e^{-\alpha \left(\frac{\lambda }{\lambda_{0}}-1\right)} & \lambda >\lambda_{0}, \end{array}\right.$$

where $\beta =\frac{1}{s_{-}}$ and $\alpha =\frac{1}{s_{+}}$. Distributions that are more leptokurtic than exponential, and/or bimodal, may provide better characterization of deleterious and beneficial mutations (Eyre‐Walker & Keightley, 2007; Levy et al., 2015); however, modeling the DFE as an exponential distribution minimizes the number of parameters in our assumptions while still capturing a distribution that has provided reasonably good fits to both deleterious (Elena, Ekunwe, Hajela, Oden, & Lenski, 1998) and beneficial (Kassen & Bataillon, 2006) fitness effects during experiments.

A new lineage with a division rate relative to the background division rate $\frac{\lambda }{\lambda_{0}}$ has probability of eventually replacing the original lineage

(4) $$p_{f\;\;i\;\;x}\left(\frac{\lambda }{\lambda_{0}}\right)=p_{f\;\;i\;\;x}\left(r\right)=\frac{1-r^{-1}}{1-r^{-X_{1}}}.$$

We use Bayes' theorem to calculate the probability density of the division rate given the mutant lineage fixed,

(5) $$\Phi \left(\lambda |\lambda_{0}\right)=\frac{p_{f\;\;\mathrm{ix}}\left(\lambda ;\lambda_{0}\right)m\left(\lambda ;\lambda_{0}\right)}{\int_{0}^{\infty }p_{f\;\;\mathrm{ix}}\left(\ell ;\lambda_{0}\right)m\left(\ell ;\lambda_{0}\right)d\ell },$$

and, redefining Equation (5) such that $f_{1}\left(\lambda \right)$ is equal to the density given the first fixation of a mutant lineage, we calculate the expected value of the division rate of this new lineage: $E\left[f_{1}\left(\lambda \right)\right]=\lambda_{1}=\int_{0}^{\infty }\lambda f_{1}\left(\lambda \right)d\lambda .$

Note that we can extend this analysis to the accumulation of mutations that do not alter the fitness of stem cells within the niche by changing Equation (4) to be equal to $\frac{1}{X_{1}}$, the probability of fixation of a neutral mutation. Additionally, mutations that affect differentiation rate can also be modeled using Equation (3) by switching the direction of effect such that beneficial mutations now decrease the differentiation rate (i.e., increase the lifetime of the cells).

#### 2.3.2 Calculating the expected effect of multiple mutations within a crypt, and the accumulation of mutations throughout all crypts

We calculate probability densities describing the division rate of *m* subsequent fixed mutations, and the probability of tumorigenesis they confer, according to the recursive formula

(6) $$f_{n+1}\left(\lambda \right)=\int_{0}^{\infty }\Phi \left(\lambda |\lambda_{0}=\ell \right)f_{n}\left(\ell \right)d\ell .$$

From Equation (6), we can calculate the expected value of division rate given *m* mutations, which is the expected value of these probability densities: $E\left[f_{m}\left(\lambda \right)\right]=\lambda_{m}=\int_{0}^{\infty }\lambda f_{m}\left(\lambda \right)d\lambda .$

We model the rate at which new lineages arise and fix in the crypts as being constant over time with rate $\hat{\mu }=\hat{p}\mu \lambda_{0}X_{1}$, where $\hat{p}$ is the total probability of fixation $\int_{0}^{\infty }p_{f\;\;i\;\;x}\left(\lambda ;\lambda_{0}\right)m\left(\lambda ;\lambda_{0}\right)d\lambda$ and μ is the mutation rate, as in Cannataro et al. (2016). Here, the number of fixed mutations within a crypt, *m*, is approximated by the Poisson distribution with mean $\hat{\mu }t$, and we can estimate the number of *n* crypts with *m* mutations by multiplying this distribution by the number of crypts in the system, *C*:

(7) $$n_{m}\approx Ce^{-\hat{\mu }t}{\left(\hat{\mu }t\right)}^{m}/m!.$$

Thus, we can estimate the total number of crypts, *n*_*m*_, with *m* mutations as the organism ages. Our model assumes that only one new lineage competes for fixation at a time, and simulations of fixation dynamics reveal that the expected number of mutations to arise during the mean time until fixation is indeed less than one for all population sizes considered here (data not shown, simulations are available at https://github.com/vcannataro/fixation_time).

#### 2.3.3 Calculating the probability that fixed mutations initiate tumorigenesis

For part of our analysis, we juxtapose the expected magnitude of tissue change with the risk of tumorigenesis for a given stem cell niche size. We define the initiation of tumorigenesis as the moment when the division rate of stem cells, λ, becomes greater than the differentiation rate of stem cells, ν, thus initiating exponential growth within the tissue. We calculate this by integrating the probability densities describing the change in the stem cell rates from ν to infinity for scenarios where mutations affect division rate, and λ to zero for scenarios where mutations affect differentiation rate, with both calculations determining the probability that a certain mutation resulted in a fitness change that initiated tumorigenesis. These probabilities are summed over all mutations in all crypts, giving the total probability of tumorigenesis.

#### 2.3.4 Calculating the expected effect of mutation accumulation on tissue maintenance

Using our calculation of the number of crypts with *m* mutations (Equation 7), the expected division rate of stem cells within niches with *m* mutations, and the steady‐state postmitotic cell population given this expected division rate (Equation 2), we can estimate the expected postmitotic epithelial population size over the time since adulthood, *t*, of the individual,

(8) $$Z^{*}\left(t\right)=Z_{\mathrm{Total}}^{*}-\sum_{m=1}^{\hat{m}}n_{m}\left(Z_{\mathrm{Normal}}^{*}-Z_{m}^{*}\right),$$

where $Z_{\mathrm{Total}}^{*}$ is equal to the total number of crypts times the number of postmitotic cells produced by a crypt with no fixed mutations, $Z_{\mathrm{Normal}}^{*}$.

Mutations that do not affect stem cell division rate fix neutrally in the stem cell niche; however, these mutations may still alter the steady‐state mean of the postmitotic terminally differentiated population by changing population dynamics rates that are important for tissue maintenance. For instance, mutations that strictly deal with the rate for a lineage to differentiate (ν) would still change Z*.

### 2.4 Linear approximation

Plots of $Z^{*}\left(t\right)/Z_{\mathrm{Total}}^{*}$ as a function of organismal lifetime are approximately linear (see Section 3.2). Hence, we utilize an asymptotic analysis to approximate the changes to the postmitotic cell population over a lifetime, that is, the dynamics defined by Equation (8). When only considering the relative affect of the first fixed mutation within stem cell niches, and the arrival of this first fixed mutation among the niches, we can simplify Equation (8) to

(9) $$\frac{Z^{*}\left(t\right)}{Z_{\mathrm{Total}}^{*}}\approx 1-\left(1-\frac{Z_{1}^{*}}{Z_{0}^{*}}\right)\lambda_{0}\mu \hat{p}X_{1}t,$$

where $\frac{Z_{1}^{*}}{Z_{0}^{*}}$ is the steady‐state population size of the postmitotic cell population after one fixed mutation divided by the healthy population size with zero mutations (Equation 2). When mutations alter the division rate, the fraction $\frac{Z_{1}^{*}}{Z_{0}^{*}}$ simplifies to $\frac{\lambda_{1}X_{1}^{*}}{\left(\nu -\lambda_{1}\right)}\times \frac{\left(\nu -\lambda_{0}\right)}{\lambda_{0}X_{1}^{*}}$, or $r_{\lambda }\times \frac{\theta -1}{\theta -r_{\lambda }}$, where $r_{\lambda }=\frac{\lambda_{1}}{\lambda_{0}}$ and $\theta =\frac{\nu }{\lambda_{0}}$. Thus, Equation (9) simplifies to $\frac{Z_{t}^{*}}{Z_{\mathrm{Total}}^{*}}=1-\left(1-r_{\lambda }\times \frac{\theta -1}{\theta -r_{\lambda }}\right)\lambda_{0}X_{1}\mu \hat{p}t$, whose time derivative is

(10) $$\frac{d}{dt}\frac{Z^{*}\left(t\right)}{Z_{\mathrm{Total}}^{*}}=\frac{-\theta \lambda_{0}\mu \hat{p}\left(r_{\lambda }-1\right)X_{1}}{r_{\lambda }-\theta }.$$

When mutations affect the differentiation rate of stem cells, the fraction $\frac{Z_{1}^{*}}{Z_{0}^{*}}$ simplifies to $r_{\nu }\times \frac{1-\theta^{-1}}{r_{\nu }-\theta^{-1}}$, where $r_{\nu }=\frac{\nu_{1}}{\nu_{0}}$. In this case, Equation (9) simplifies to $\frac{Z_{t}^{*}}{Z_{\mathrm{Total}}^{*}}=1-\left(1-r_{\nu }\times \frac{1-\theta^{-1}}{r_{\nu }-\theta^{-1}}\right)\lambda_{0}\mu t$, and the derivative of this function with respect to time is as follows:

(11) $$\frac{d}{dt}\frac{Z^{*}\left(t\right)}{Z_{\mathrm{Total}}^{*}}=\frac{-\mu \lambda_{0}\left(r_{\nu }-1\right)}{\theta r_{\nu }-1}.$$

Note how Equation (11) is independent of *X*_1_ and $\hat{p}$, as mutations fix neutrally and these terms are the inverse of one another.

Equations (10) and (11) are close approximations of the rate of tissue size change per day. Importantly, they are independent of many of the parameters in the crypt model (1), such as the division rate and number of generations of TA cells and the apoptosis rate of postmitotic cells. These parameters are not easily measured in humans, and thus, Equations (10) and (11) enable us to extend our inference to human scenarios.

#### 2.4.1 Parameterization for the human colon

The division rate of stem cells within human colon crypts has been reported as close to once a week (Nicolas, Kim, Shibata, & Tavaré, 2007; Potten, Booth, & Hargreaves, 2003), and the total number of stem cells maintaining the crypt population, that is, stem cells in the niche, has been estimated to be between six (Baker et al., 2014) and 15–20 cells (Nicolas et al., 2007), with Bayesian analysis from the latter study showing more support for larger crypt population sizes. Assuming the human stem cell niche in the colon crypt houses six cells, and the total mean number of potential stem cells in the colon crypt is approximately 36 cells (Bravo & Axelrod, 2013), we use Equation (1) to calculate that the differentiation rate of stem cells is approximately 0.172 per day. Alternatively, if the human stem cell niche houses 20 cells, and there are a total of 36 putative stem cells within the crypt, we calculate the differentiation rate of stem cells to be 0.321 per day. Thus, we have all the parameters necessary to calculate the expected effect of the accumulation of mutations in stem cell niches on tissue homeostasis in human intestines, and can compare the expected effects given the two different estimated sizes of the stem cell niche.

### 2.5 Exploring the evolutionary trade‐off between stem cell niche size, aging, and tumorigenesis

Given that small asexual populations are prone to succumb to a gradual decline in mean fitness via the accumulation of deleterious mutations (Lynch et al., 1993), we explore the selective pressures that may have influenced the evolution of small stem cell niche population size. Specifically, we juxtapose the magnitude of epithelium tissue population change and the total risk of tumorigenesis throughout the intestinal epithelium for different stem cell niche sizes. Throughout this analysis, we assume that half of the putative stem cells in the crypt reside in the niche, as is approximately the case for both mice and humans. Additionally, we emphasize that the absolute magnitudes of tissue change and tumor incidence may not reflect the true values, as the parameters associated with the true expected mutational effects are unknown; however, the emergent selective dynamics from our analysis are relatively independent of exact parameter specifications.

## 3 Results

### 3.1 The expected fitness of stem cells decreases with fixed mutations

#### 3.1.1 When mutations affect division rate

**Figure 2:**
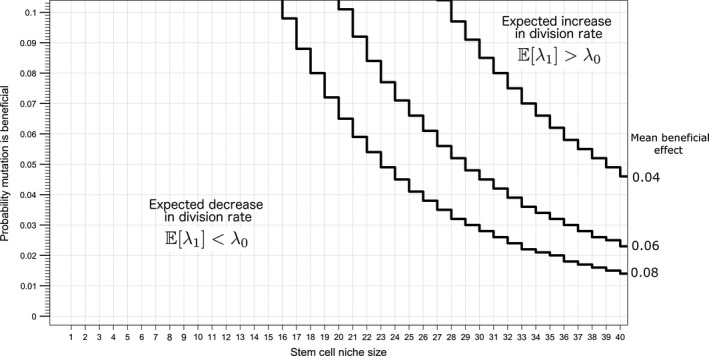
Expected values of division rate after a fixed mutation are lower than the original division rate (deleterious) for reported stem cell niche sizes and plausible DFE parameters. The lines plotted here represent the boundary in the parameter space that separates the scenarios where beneficial or deleterious mutations are expected to accumulate. The mean deleterious effect for the parameter space depicted here is 0.15

The small population size of the stem cell niche at the base of the crypts promotes weak selection and pervasive drift throughout the intestine. For each crypt, across a range of distribution of fitness effect parameters consistent with those measured in whole organisms and stem cell niche sizes consistent with those measured in mice and humans (between 6 and 20 cells), the expected value of the first fixed mutation has a lower fitness than the previous lineage when mutations affect division rate (Figure 2). The expected value of a fixed mutation that affects division rate increases with larger values of population size, the expected effect of a beneficial mutation (*s*_+_), and the probability that a mutation results in a beneficial effect.

**Figure 3:**
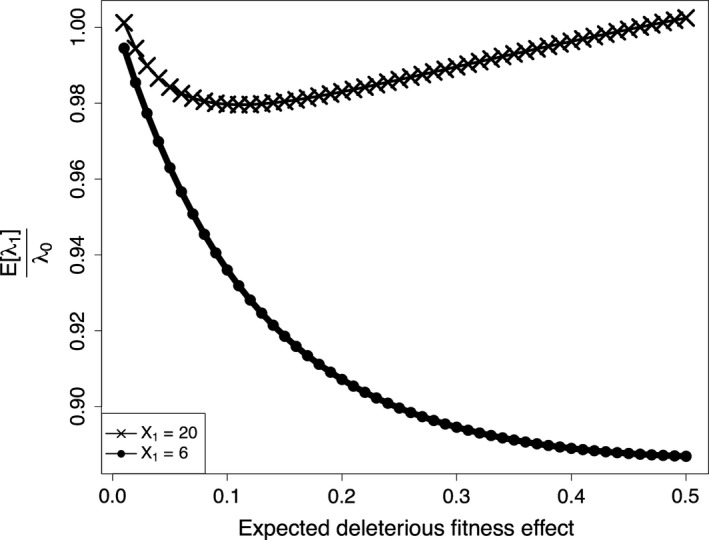
The expected value of division rate divided by the original division rate, $r_{\lambda }$, versus $s_{-}$, the expected mutational effect given a deleterious mutation, for the stem cell niche sizes of 20 and 6. Here, $s_{+}$ is 0.061 and *P*_B_ is 0.0575

The weak selective pressure at small populations is exhibited by considering the expected effect of a fixed mutation as a function of the expected effect of deleterious fitness effects at two population sizes (Figure 3). For smaller populations, such as when *X*_1_ = 6, even mutations of large deleterious effect may fix through genetic drift, and then the expected value of the first fixed mutation continues to decrease as the expected value of a deleterious mutation increases. Alternatively, larger stem cell niche population sizes are less prone to deleterious mutations fixing through drift, and as the expected effect of deleterious mutations increases, there is a point at which deleterious mutations become less and less likely to fix. Given a fixation event, it is likely the mutation conferred a beneficial effect.

#### 3.1.2 When mutations affect differentiation rate

As the differentiation rate phenotype is not expressed in the stem cell niche, all mutations to this rate fix neutrally, and the expected value of ν given a fixed mutation does not depend on niche population size. Because the vast majority of mutations are deleterious, the expected value of mutations is always deleterious in the absence of selection.

### 3.2 Mouse and human stem cell fitness is expected to decrease with age, reducing tissue renewal

**Figure 4:**
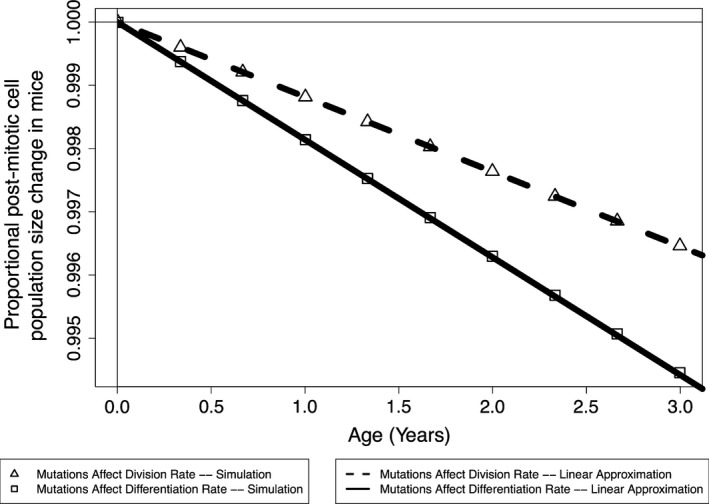
The expected tissue size change in mice due to mutation accumulation

**Figure 5:**
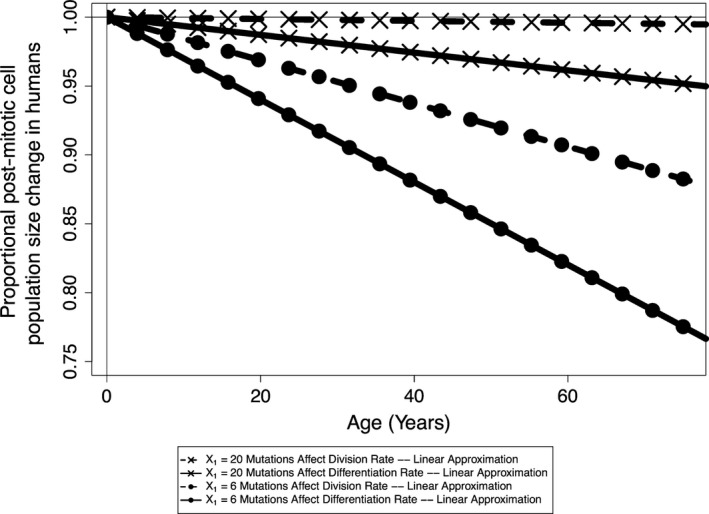
The expected tissue size change in humans due to mutation accumulation

As demonstrated in Section 3.1, the fitness of stem cells is expected to decrease as mutations accumulate in stem cell niches. This results in diminished whole‐tissue population sizes as mutations accumulate throughout the crypts within the tissue in mice (Figure 4) and humans (Figure 5). We find that the linear approximation described in Section 2.4 provides a good approximation to the simulated curves, and we thus employ this approximation in estimating tissue size change curves for humans. Interestingly, when considering mutations that affect division rates in mice and humans (assuming a human stem cell niche population size of 20), we predict similar postmitotic cell population size changes throughout organism lifetimes, with population size declining approximately 0.35% and 0.5%, respectively. If we assume that the population size in the human stem cell niche is 6 and mutations only affect division rate, we predict a decline in population size of approximately 12% over human lifetime. When mutations only affect differentiation rate, we predict larger declines in population size, as there is no selective pressure against the fixation of deleterious mutations in this scenario.

### 3.3 There is an evolutionary trade‐off between tumorigenesis and aging mediated by stem cell niche size

**Figure 6:**
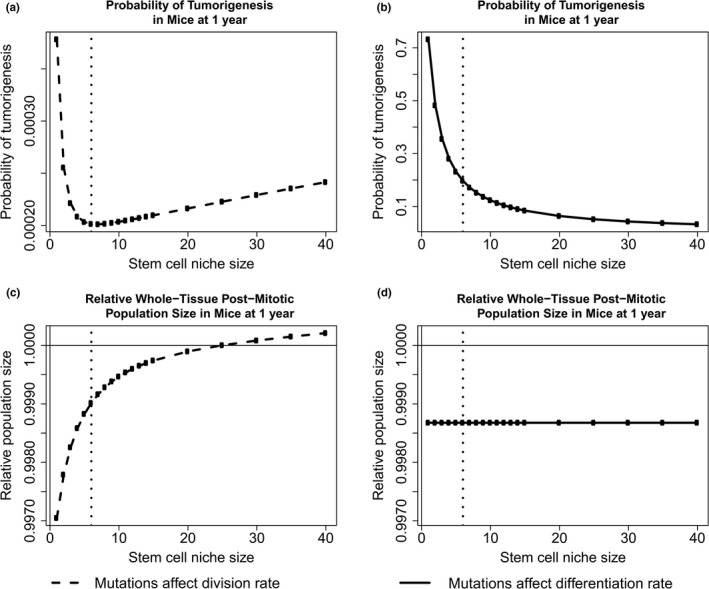
An evolutionary trade‐off with niche size in mice. For scenarios where mutations affect division rate, tumorigenesis is minimized at an intermediate population size (a), at the expense of fixing deleterious mutations and decreasing tissue renewal (c). For scenarios where mutations affect differentiation rate, tumorigenesis is minimized for large population sizes (b), and the effect of mutation accumulation on the epithelium is independent of niche size (d). The black vertical dotted line is placed at 6, the median niche size measured in the mouse

**Figure 7:**
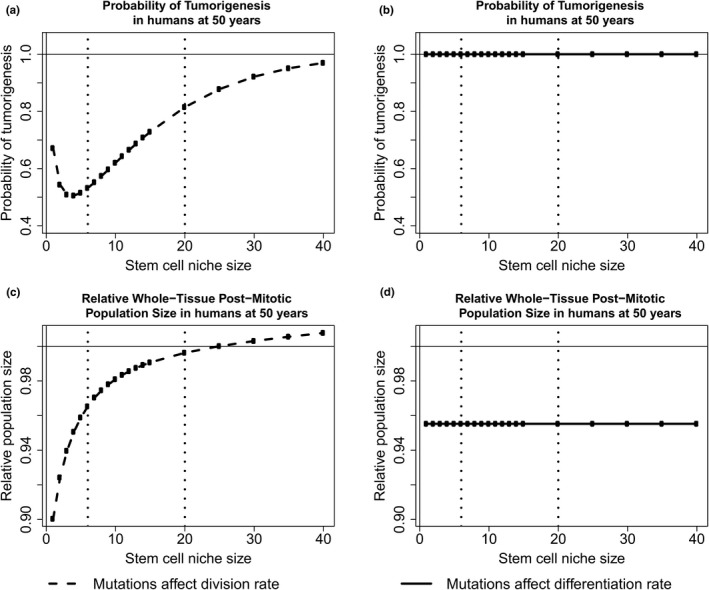
An evolutionary trade‐off with niche size in humans. For scenarios where mutations affect division rate, tumorigenesis is minimized at an intermediate population size (a), at the expense of fixing deleterious mutations and decreasing tissue renewal (c). For scenarios where mutations just affect differentiation rate, tumorigenesis is minimized for large population sizes (b), and the effect of mutation accumulation on the epithelium is independent of niche size (d). The black vertical dotted lines are placed at 6 and 20, the reported stem cell population sizes in humans

We vary initial stem cell population size, along with the total number of crypts in the system, such that the total output of crypts remains constant. This analysis was initially conducted using the crypt parameters described for the mouse in Table 1 and the yeast mutation parameters described in Section 2.2, namely mutations that affect fitness occur at rate 1.26 × 10^−4^, beneficial fitness effects have an expected value of 0.061, deleterious effects have an expected value of 0.217, and 5.75% of mutations confer a beneficial fitness effect. For mutations that affect division rate, there exists an optimal intermediate crypt size to minimize the probability of tumorigenesis (Figure 6a). However, at this crypt size, the expected value of the epithelium tissue size is expected to decrease over a lifetime due to the accumulation of deleterious mutations in stem cell niches (Figure 6c). Furthermore, when mutations affect differentiation rate and fix neutrally, the probability of tumorigenesis is minimized for large stem cell niche sizes (Figure 6b) and the expected effect on tissue size is invariant to stem cell niche size (Figure 6d). As the true mutational parameters governing somatic tissue evolution are unknown, we do not wish to analyze the predicted magnitude of tumorigenesis and aging presented here, but rather the trade‐off that exists between niche population size, drift, and selection among mutations that confer a selective advantage and those that fix neutrally. The nature of the dynamics presented in Figure 6 holds true for a large range of mutation parameters. For instance, we next analyzed this evolutionary trade‐off when considering the parameters discussed in Section 2.4 for the human colon (Figure 7). We find that the probability of a tumorigenesis event is minimized at a similarly small population size when mutations affect division rate, and there is a higher probability of tumorigenesis and larger change in population size for all human scenarios when compared to the results from the mouse model.

## 4 Discussion

### 4.1 The effects of mutational target on optimal stem cell niche size

When mutations confer a selective advantage or disadvantage within the niche, there exists an intermediate crypt size that minimizes the probability that any crypt accumulates the large beneficial mutations necessary to initiate a tumor. This result reinforces previous results by Michor et al. (2003), who also found an intermediate niche size exists that decreases the rate of tumor initiation for mutations under selection. By modeling the fixation of mutations drawn from a full distribution of mutational effects and accumulating throughout the populations of the entire intestinal epithelium, we show that a secondary trade‐off exists—populations maintained at a size that results in the lowest rate of tumorigenesis are expected to accumulate deleterious mutations that manifest in tissue attrition and contribute to organismal aging.

At small stem cell niche sizes, there exists a large number of crypts to maintain homeostasis, and a higher probability that any one crypt will obtain a rare mutation of large effect that would result in tumorigenesis. As stem cell niche size increases, the number of crypts needed to maintain the same amount of epithelium decreases, and so does the probability of fixing mutations within the crypts, and therefore the chance of fixing a rare mutation of large effect. However, for larger values of stem cell niche size, the strength of selection increases, thus increasing the chance that a fixed mutation was beneficial, leading to higher chances of tumorigenesis. Here, given the specified parameters for mice, we find that the minimum probability of tumorigenesis exists at seven cells, which is within the empirically reported stem cell number (Kozar et al., 2013; Ritsma et al., 2014) for intestinal stem cell niches in mice. At this population size, the whole tissue size is expected to decline with age as deleterious mutations accumulate in stem cell niches, and if selective pressures against tumorigenesis have selected for stem cell niche population sizes at this intermediate, then it has been at the expense of increasing epithelial attrition. When considering parameters reported for the human intestinal epithelium and mutations that affect division rate, we find that the population size with the smallest probability of tumorigenesis is lower than the reported sizes. We note that the experimental methods used to elucidate stem cell population size in mice are not possible in humans, and understanding the true size of the stem cell niche in larger, long‐lived animals will shed light on how long‐lived organisms balance maintaining their tissues while minimizing mutation‐associated disease. Furthermore, we find that the probability of tumorigenesis is higher for humans when compared to mice, as one might expect given that humans live longer and have more cells (i.e., opportunities for cancer initiation) than mice. However, cancer incidence is not directly correlated with body mass in nature, a phenomenon known as Peto's Paradox, pointing to the potential existence of mechanisms in long‐lived large animals that may suppress cancer formation and growth (Caulin & Maley, 2011).

When mutations affect differentiation rate and thus fix neutrally in the stem cell niche, larger stem cell niche sizes result in a lower probability of tumorigenesis. However, the rate at which the epithelium changes total size as mutations accumulate within stem cell niches is independent of stem cell niche size. At small niche population sizes, the number of fixed mutations in the entire system is maximized as both the number of crypts and probability of fixation of every mutation within each crypt are maximized, thus leading to the maximum probability that any one fixed mutation resulted in tumorigenesis. Because these mutations fix neutrally, the expected value of a fixed mutation is independent of stem cell niche population size. Furthermore, the probability of fixation of a mutation in a niche is the inverse of the contribution of that niche to tissue homeostasis. For instance, a mutation that arises within a niche that is ten times the size of another niche has ten times smaller probability of fixation, but ten times higher influence on the total epithelium, meaning that the expected influence of the total amount of accumulated mutations in systems with different stem cell population sizes but consistent total epithelium sizes is invariant.

The results discussed above were obtained using a single biologically informed distribution of fitness effects as we are primarily concerned with exploring the selection pressures on stem cell niche population size. We have previously explored the implications that different distributions of fitness effects have on tumorigenesis incidence in mice and humans (Cannataro et al., 2016). The distribution of fitness effects influences the rate of tissue size change in the model presented here by affecting the expected value of the effect within the first fixed mutated lineage within a crypt and thus will affect $r_{\lambda }$ in Equation (10), and $r_{\nu }$ in Equation (11), and $\hat{p}$ in both equations. Specifically, as the expected value of the division rate of fixed mutations increases, tissue attrition with age decreases and the probability of tumorigenesis increases. For the differentiation rate scenarios, tissue attrition decreases and tumorigenesis increases as the expected value of the differentiation rate of fixed mutations decreases.

It is possible that the mutational target size for mutations that affect the propensity for stem cells to commit to differentiation is smaller than that for mutations that might affect the overall division rate, and therefore, selection may not act as strongly to optimize niche size in light on minimizing tumorigenesis caused by failure to differentiate. Furthermore, our model results indicate that both the probability of tumorigenesis and the extent of tissue size change are larger for the scenarios where mutations only affect differentiation rate. This is due to mutations in this scenario fixing neutrally and having a larger absolute influence toward tumorigenesis (we model expected mutational effect sizes as a proportion of the rate they govern). We use the same rate of mutation when modeling both mutations that affect division rate and differentiation rate, but if there were a smaller target size for mutations that change the rate at which stem cells commit to differentiation, there would also be a smaller mutation rate. In addition, we chose to model the accumulation of mutations that affect division and differentiation rates as separate scenarios to minimize the number of unknown parameters in our model, but mutations to certain genes, such as APC, may affect both rates (McCartney & Näthke, 2008).

We utilize an analytical approximation of cells replacing neighbors in a one‐dimensional ring, which is a proposed method of stem cell turnover (Lopez‐Garcia et al., 2010; Vermeulen et al., 2013), for the probability of fixation of new mutations within the stem cell niche. Certain spatially explicit models of stem cell niches, such as bowl‐like network graphs and weighted graphs, result in less selective pressure on new mutations when compared to our model and lead to higher probabilities of fixation for deleterious mutations and lower probabilities of fixation for beneficial mutations (Hindersin, Werner, Dingli, & Traulsen, 2016). Thus, the estimates presented here of tumorigenesis may be higher and the estimates of tissue attrition may be lower when compared to more complex spatially explicit models.

#### 4.1.1 The intestinal epithelium population is expected to decline with age through stem cell attrition

When employing distributions of mutational effects commonly found in experiments on whole organisms, we find that the expected effect from mutation accumulation alone is a decrease in the total intestinal epithelium size with age. This attrition is modest in the mouse, with a mouse 3 years into adulthood having an intestinal epithelium approximately 0.3%–0.5% smaller than a mouse that just entered adulthood. This attrition is potentially much more substantial for humans, given their longer lifetime. When mutations affect division rate and for the scenario where the stem cell niche size exists as 20 cells, we find remarkably similar total epithelial population change to the mouse scenario because, despite the longer human lifetime, the division rate of stem cells is slower and the population size is larger, increasing the strength of purifying selection and decreasing the rate at which deleterious mutations accumulate. When mutations fix neutrally for the same population size and affect differentiation rate, we calculate that the total postmitotic epithelium will decrease approximately 5% at 75 years postadulthood. If the true stem cell niche size in humans is closer to the lower estimate of six cells, we calculate a much higher rate of tissue attrition, with the epithelium decreasing over 10% and over 20% in the division and differentiation scenarios, respectively. This is due to the much stronger influence of deleterious mutations drifting to fixation at smaller population sizes. Empirically, both mitotic activity of intestinal cells and intestinal weight relative to total body weight gradually decline with age in adult rats (Mandir, FitzGerald, & Goodlad, 2005), although the causative factors of these dynamics have not been elucidated. We recognize that this model only considers the effects of mutation on cell replacement rates and does not consider what concomitant effects might mitigate or even exacerbate these effects. More empirical studies of the intestine with age are needed to understand the importance of mutation accumulation for health and aging overall.

#### 4.1.2 Mechanisms of intestinal homeostasis and evolutionary trade‐offs

Although mice and humans have an intrinsic rate of crypt division, this event is exceedingly rare (Baker et al., 2014; Snippert et al., 2014) and likely does not play a role in maintaining tissue homeostasis. Thus, population equilibrium during normal tissue maintenance is primarily controlled through the continual division and differentiation of stem cells. We emphasize here that our estimated decline is solely the expected effect from mutation accumulation within crypts, with no other potential (and unknown) mechanisms to compensate for decreased division potential. Lineage tracing experiments in mouse adenomas reveal that oncogenically activated (i.e., cellular fitness increased by mutation) epithelial cells retain their crypt‐like structure and have larger number, but a similar proportion, of stem cell marker‐expressing cells (Schepers et al., 2012). Similarly, we assume that crypts containing mutations that decrease fitness will maintain crypt population dynamics while having decreased stem cell, and total cell, population numbers. Here, we show that, due to the small population sizes of the independently evolving populations of stem cells constituting the intestinal epithelium, mutations are expected to contribute to a decrease in epithelial renewal, and total size, over an organism's lifetime. Furthermore, we have demonstrated that the intestinal stem cell compartment is an example of Muller's ratchet and mutational meltdown, or, the gradual decline in fitness and population size associated with small asexual populations (Lynch et al., 1993). These small compartments minimize the overall probability that any mutation in the intestines results in the origin of a tumor when mutations confer a selective advantage in the niche, and it is thus a prime example of an evolutionary trade‐off.

## Data Archiving Statement

All data used in this manuscript have already been published or archived elsewhere.
